# macJNet: weakly-supervised multimodal image deformable registration using joint learning framework and multi-sampling cascaded MIND

**DOI:** 10.1186/s12938-023-01143-6

**Published:** 2023-09-19

**Authors:** Zhiyong Zhou, Ben Hong, Xusheng Qian, Jisu Hu, Minglei Shen, Jiansong Ji, Yakang Dai

**Affiliations:** 1 Suzhou Institute of Biomedical Engineering and Technology, Chinese Academy of Sciences, Suzhou, Jiangsu China; 2 School of Electronic and Optical Engineering, NanJing University of Science and Technology, Nanjing, Jiangsu China; 3 School of Biomedical Engineering (Suzhou), Division of Life Sciences and Medicine, University of Science and Technology of China, Suzhou, Jiangsu China; 4 Key Laboratory of Imaging Diagnosis and Minimally Invasive Intervention Research, The Fifth Affiliated Hospital of Wenzhou Medical University, Lishui, Zhejiang China

**Keywords:** Deformable registration, Multimodal, Image descriptor, Joint learning, Semi-supervised segmentation

## Abstract

Deformable multimodal image registration plays a key role in medical image analysis. It remains a challenge to find accurate dense correspondences between multimodal images due to the significant intensity distortion and the large deformation. macJNet is proposed to align the multimodal medical images, which is a weakly-supervised multimodal image deformable registration method using a joint learning framework and multi-sampling cascaded modality independent neighborhood descriptor (macMIND). The joint learning framework consists of a multimodal image registration network and two segmentation networks. The proposed macMIND is a modality-independent image structure descriptor to provide dense correspondence for registration, which incorporates multi-orientation and multi-scale sampling patterns to build self-similarity context. It greatly enhances the representation ability of cross-modal features in the registration network. The semi-supervised segmentation networks generate anatomical labels to provide semantics correspondence for registration, and the registration network helps to improve the performance of multimodal image segmentation by providing the consistency of anatomical labels. 3D CT-MR liver image dataset with 118 samples is built for evaluation, and comprehensive experiments have been conducted to demonstrate that macJNet achieves superior performance over state-of-the-art multi-modality medical image registration methods.

## Introduction

Multimodal medical image registration aims to establish anatomical correspondences between multimodal images, which plays an important role in assisted diagnosis, image-guided ablation, and surgical navigation. Medical image registration is a high-dimensional optimization task to estimate the dense deformation fields. With the recent advances in data driven learning, deep learning-based registration methods have achieved comparable accuracy with a significantly higher inference speed. In general, deep learning-based registration could be categorized into fully-supervised registration, unsupervised registration and weak-supervised registration from the perspective of the utilization of the ground-truth.

### Fully-supervised registration

Inspired by the FlowNet for vector flow estimation [1], fully-supervised image registration methods [2–4] consider image registration as a regression problem to predict deformation fields for matching the ground-truth. Fully-supervised registration imports image pairs and dense correspondence to learn the spatial mapping between images, and directly predicts deformation fields in the inference stage. It makes the fully-supervised registration a modality-independent registration method. However, it is challenging to find the accurate dense correspondence between medical images. Fan et al. [5] proposed brain image registration networks (BIRNET) to guide the training process in fully-supervised learning using a dual supervision loss to measure the difference between the generated deformation field and the real deformation field. Cao et al. [6] cascaded Syn [7] and Demons [8] to obtain the deformations used as ground-truth for CNN training. Some methods generate the artificially synthesized images to simulate the deformation fields [9], which solves the problem of getting dense correspondence between images. However, the authenticity problem of synthesized warped images would degrade registration performance.

### Unsupervised registration

Unsupervised registration methods do not require ground-truth deformation fields [10, 11], which consider image registration as a loss function minimization problem and use a differentiable warping module with the spatial transformer network (STN) [11] to warp the moving image in the training procedure. The image similarity metric and regularization are usually incorporated into the loss function to optimize the registration network. Learning the cross-modality representation through network training or designing elaborated modality-independent similarity metrics are two alternative ways for multimodal registration.

In the first way, Balakrishnan et al. [11] proposed the first unsupervised learning registration method (VoxelMorph) for mono-modality registration. Mok and Chung [12] further improved its performance by adding the symmetric diffeomorphic properties into the network. To efficiently train a medical image registration network, DeepFLASH [13] computes the deformation fields via utilizing low-dimensional band-limited space. Yan et al. [14] first proposed the adversarial image registration framework, which performs image registration tasks through a generator and evaluates the quality of the warped images by a discriminator. Kim et al. [15] proposed a fully convolutional self-similarity to find dense semantic correspondence in mono-modality registration. A recent trend for multimodal image registration takes advantages of image to image translation [16], generative adversarial networks (GANs) convert the multimodal registration into a simpler unimodal task by learning transferable representations from multimodal images. Fan et al. [17] further extended this work to both unimodal and multimodal registration. However, image translation is a challenging topic by itself, the main challenges for GANs-based registration include: it may inevitably produce artificial features [18] and achieving Nash equilibrium in training procedure [19].

In other way, some methods attempt to elaborately design cross-modal descriptors as a similarity metric to represent the modality-independent structure features for multimodal registration. Schechtman and Irani [20] introduced the local self-similarity (LSS) descriptor for multimodal image matching address the problem of multimodal appearance and shape change. Heinrich et al. [21] proposed a modality-independent neighborhood descriptor (MIND) based on self-similarity theory [20], which calculates the difference between patches within a local neighborhood. Some other LSS-based methods are also introduced to represent the cross-modal dense correspondence [22, 23]. Kim et al. proposed deep self-correlation (DSC) [24] to estimate cross-modal dense correspondences inspired by LSS and DSC has demonstrated its high accuracy on aligning multimodal image. Fully convolutional self-similarity (FCSS) [15] formulates LSS within a fully convolutional network to simultaneously learn the patch sampling patterns and self-similarity measures. Although FCSS dramatically improved performance for object-level semantic correspondence, it cannot deal with complex geometric variations, which frequently appears in medical image registration.

### Weakly-supervised registration

Weakly-supervised registration usually uses anatomical segmentation labels as semantic prior information to improve the registration performance. However, manual delineation of anatomical labels is a time-consuming and laborious work. To address the problem of insufficient labels, the joint learning framework for registration and segmentation has been proposed [25–27], in which the registration and segmentation network are alternately optimized during the training procedure. Some label-driven weakly-supervised methods have also been proposed [28, 29] by exploiting the auxiliary anatomical information and the invertible transformation. In the joint learning framework, the anatomy labels created by the segmentation network provide semantic prior knowledge to guide dense correspondence mapping for the registration network [25]. The registration provides the consistency of segmentation labels by mapping the warped image to the fixed image, which is an effective way to improve the segmentation performance of multimodal images. The registration and segmentation networks are iteratively optimized in an end-to-end manner to simultaneously improve the performances of registration and segmentation [30]. However, the joint learning framework still confronts the following problems. For registration network, it is a challenge on how to utilize semantic labels to provide sufficient dense correspondence between multimodal images [31], which leads to the low quality of registration in interior of large tissues, such as liver. For segmentation network, it is a challenge to generate the consistent labels for multimodal images with few manual labels.

In general, the existing registration methods cannot accurately align the multimodal images since they cannot learn the cross-modality dense correspondence to handle complex and large deformation. In this paper, macJNet is proposed as a novel multi-modality registration method, which is weakly-supervised multimodal image deformable registration using joint learning framework and multi-sampling cascaded modality independent neighborhood descriptor (macMIND). The key idea behind macJNet is to learn (or extract) different levels of prior knowledge to guide the registration: anatomical labels are predicted by segmentation networks as semantic information to provide global sparse correspondences for registration, and the macMIND is extracted as context information to provide local dense correspondences for registration. Our contributions are summarized as follows.

1. A novel weakly-supervised multimodal image deformable registration methodology using a joint learning framework (macJNet) is proposed for multimodal registration. The macJNet consists of a registration network and two segmentation networks, which are iteratively optimized in a single end-to-end framework. Segmentation networks provide semantic anatomical labels for weakly-supervised registration by few-label learning; registration network improves the performance of segmentation results by enforcing cross-modality consistency based on deformable spatial mapping.
2. Multi-sampling cascaded modality independent neighborhood descriptor (macMIND) is proposed to establish dense correspondences between multimodal images for registration. macMIND builds the local self-similarity context by multi-orientation and multi-scale sampling in a supporting window, which enriches the modality-independence contextual information to characterize cross-modality anatomical structures. An efficient computational scheme for macMIND in a convolutional manner is also proposed.
3. Dual similarity-based loss function is introduced to optimize macJNet. The dual similarity incorporates macMIND and DSC, in which macMIND represents the similarity of modality-independent context to find dense correspondence and DSC represents the similarity of semantic labels structures to find sparse correspondence of tissue boundaries.

The paper is organized as follows. “Experiments” presents the proposed methodology and its implementation. The experiments results are given in “Methodology”. Conclusion and discussion are given in "Methodology".

## Experiments

### Medical image data and evaluation metrics

**Fig. 1:**
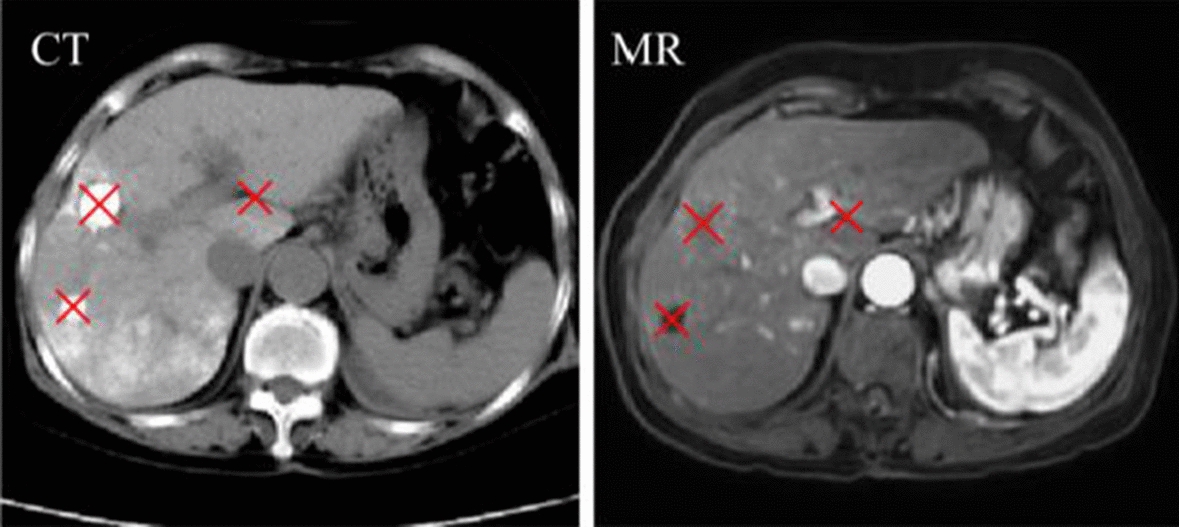
The identified landmarks (the central location of tumor and hepatic fissures) in CT and MR images. The TRE is defined as Euclidean distance between the corresponding landmarks

118 pairs of CT-MR liver images are used to evaluate the proposed method. All images are collected from Lishui Central Hospital. The characteristic of dataset is listed in Table 1. All anatomy labels (liver labels and tumor labels) and anatomical landmarks (Fig. 1) are executed by radiologists. 90 pairs are selected randomly and assigned into training cohort, and the remaining 28 pairs are assigned into the testing cohort. macJNet is optimized by five-fold cross-validation on the training cohort.

**Table 1:** The characteristic of the dataset

Symbol	MR image	CT image
Modality	T1(no contrast)	no contrast
FOV	288 × 288	512 × 512
Resolution (mm^3^)	1.146 × 1.146 × 3	0.664 × 0.664 × 5
Scanner	Siemens	Philips

To quantitatively verify the effectiveness of macJNet, target registration error (TRE), Dice similarity coefficient (DSC), 95% Hausdorff distance (Hd_95_), mutual information (MI), and structural similarity (SSIM) are used to evaluate the registration accuracy. TRE, DSC and Hd_95_ are used to evaluate the accuracy of tumor and liver registration; MI and SSIM are used to evaluate the registration quality over the entire image domain.

Mutual information is a common similarity metric for multimodal image registration, which indicates the similarity of two images. The mutual information is defined as:

1 $${\text{MI}}\left( {I_{F} ,I_{M} } \right) = \sum\limits_{{I_{F} ,I_{M} }} {p\left( {I_{F} ,I_{M} } \right)\log \frac{{p\left( {I_{F} ,I_{M} } \right)}}{{p\left( {I_{F} } \right)p\left( {I_{M} } \right)}}} ,$$

where the probability *p*(*I*) is the probability distribution of the voxel values in image *I*, and the probability *p*(*I*_*F*_,* I*_*M*_) is the joint distribution of the intensities of two images.

SSIM is a metric to measure the structural similarity between two images, which mainly focus on structural information (such as shapes and position). The range of SSIM is from 0 to 1, a higher value implies a higher similarity [32]. SSIM has been applied as similarity metric in a GAN-based brain multimodal registration [33]. SSIM is defined as

2 $${\text{SSIM}} = \frac{{\left( {2\overline{I}_{F} \overline{I}_{M} + c_{1} } \right) + \left( {2\sigma_{M - F} + c_{2} } \right)}}{{\left( {\overline{I}_{F}^{2} + \overline{I}_{M}^{2} + c_{1} } \right)\left( {\sigma_{F}^{2} + \sigma_{M}^{2} + c_{2} } \right)}},$$

where $\overline{I}$ symbolizes the mean voxel value of the given image; *σ* is the standard deviation of the image; *σ*_*M*-*F*_ is the covariance of multimodal image pair; *c*_1_ and *c*_2_ are constant values.

### Registration results

#### Implementation

In light of the limited GPU computing resources, the liver images are resampled into 128 × 128 × 96 and then input into macJNet for training and inference. The output deformation fields and warped images would be up-sampled to original size. The Reg-SubNet is pre-trained in an unsupervised manner, and Seg-SubNets is pre-trained in cycle self-training with CT and MR image. 30% liver labels are used to train the Seg-SubNets for guiding registration, and the tumor labels are only used as ground-truth to evaluation the accuracy of registration. The learning rate is set to 2 × 10^−5^ in registration and 1 × 10^−5^ in segmentation, batch size is 1, epoch number is 200. The learning rate in registration network is larger than that in segmentation due to the convergence of segmentation is faster than registration. Adam is used as optimizer in these networks. In our experiments, the hyper-parameters are: *K* = 2, *α*_1_ = 0.3, *α*_2_ = 0.7 in Eq. (11); *λ*_sim_ = 20, *λ*_label_ = 2 and* λ*_smo_ = 0.5 in loss function. *L* = 2-pixel distance, *R*_1_ = *R*_2_ = 5-pixel. The joint training cost around 16 h to reach convergence, while it only cost about 0.18 s to complete deformation prediction for an image pair.

To evaluate the registration performance, macJNet is performed to compared with the well-performed methods: Elastix [34], VoxelMorph [11], and LapIRN [35]. Elastix is a classic traditional registration method using mutual information-based multimodal similarity metric, and 3-level pyramid in Elastix is used in the experiments. VoxelMorph is a CNN-based unsupervised registration method, which is aimed to mono-modality image registration. VoxelMorph with MIND-based loss function is applied to multimodal registration. The configuration of VoxelMorph is as follows: learning rate of 1 × 10^−4^, regularization parameter of 1, batch size of 1, and the number of epochs of 800. LapIRN is a CNN-based registration method, which divides the image into three resolutions and performs registration layer by layer. LapIRN is also applied as a baseline network in Reg-SubNet. The parameters of configuration are set same as VoxelMorph. All deep learning-based methods are implemented by Pytorch on a single Nvidia Telsa V100 GPU with 16G memory. Elastix registration running on AMD Ryzen 5 4600H CPU. Affine alignment for each image pair is pre-performed using Elastix to reduce the position deviation.

#### Multimodal image registration results

**Fig. 2:**
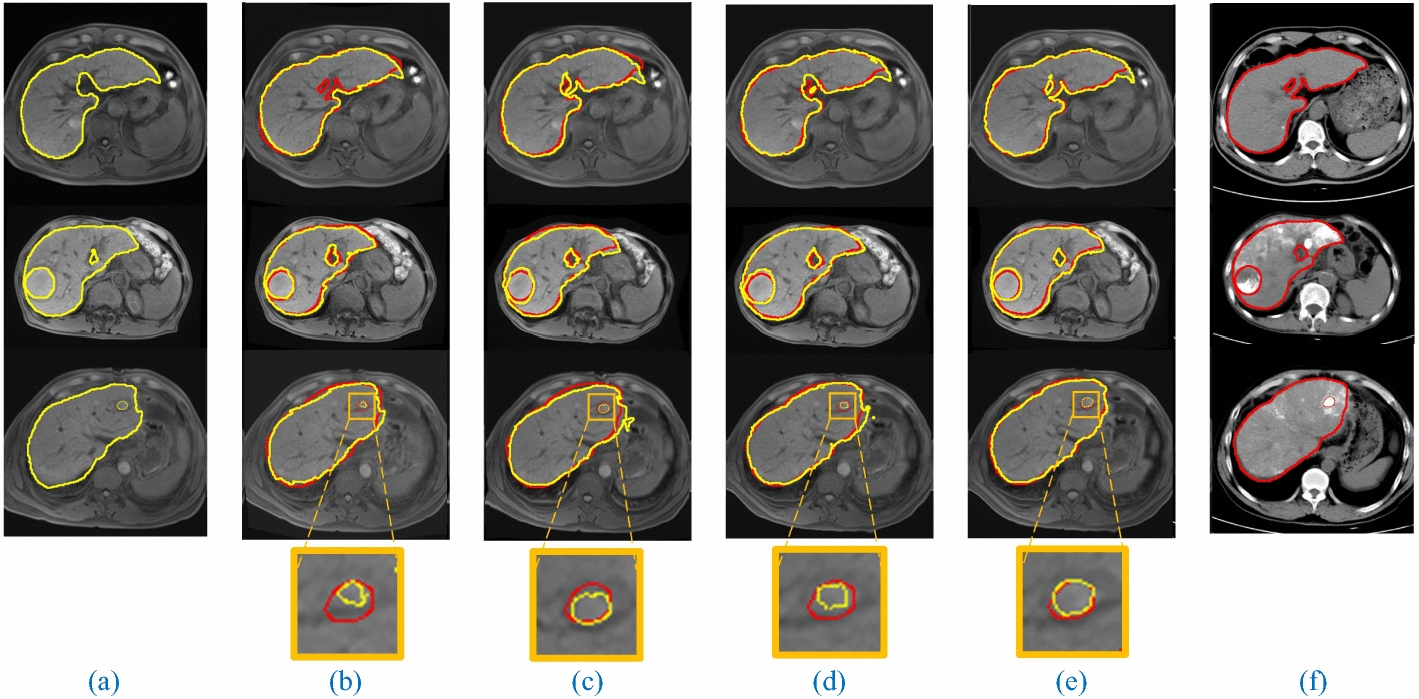
Visualization of registration on three samples in the test dataset. The left and right columns show the moving and fixed images, respectively. The middle four columns show the results of Elastix, VoxelMorph, LapIRN and macJNet in sequence. **a** MR image (moving image), **b** registration results of Elastix, **c** registration results of VoxelMorph, **d** registration results of LapIRN, **e** registration results of macJNet, **f** CT image (fixed image)

As shown in Table 2, four deformable registration methods are compared with the metrics of TRE, DSC, Hd_95_, MI, SSIM and inference time. In terms of tumor registration, it is observed that macJNet achieves better registration performance (TRE = 5.05 mm, DSC = 55.20%, Hd_95_ = 6.71 mm) than Elastix, VoxelMorph and LapIRN. In terms of liver registration, macJNet (TRE = 4.83 mm, DSC = 94.75%, Hd_95_ = 4.53 mm, MI = 44.12%, SSIM = 54.43%) also outperforms other competitive methods in all evaluation metrics. This statistical result demonstrates that macMIND and consistency constraint simultaneously improve the global registration accuracy and local accuracy. Figure 2 intuitively shows the visual comparisons of registration results using different methods, where macJNet optimizes the deformation both in tissue boundary and internal organs. The Elastix outperforms VoxelMorph at tumor alignment with the metric of TRE and DSC and liver alignment with all evaluation metrics. In addition, the inference time of macJNet is comparable to other deep learning-based methods and over 400 times faster than Elastix. The affine registration is listed as a reference to obviously compare the performance of registration methods. It should be noted that clinical medical images are used (slice thickness is larger than 3 mm) in the experiment, which takes an adverse impact on registration result. However, macJNet still accurately matches the multimodal images, and outperforms the competitive methods.

**Table 2:** Comparisons of registration results (mean ± std)

Methods	Tumor			Liver			Image		Time (s)
	TRE (mm)	DSC (%)	Hd_95_ (mm)	TRE (mm)	DSC (%)	Hd_95_(mm)	MI (%)	SSIM (%)	
Affined	7.38 ± 3.56	46.81 ± 32.67	7.70 ± 3.27	7.01 ± 2.95	90.94 ± 1.38	6.67 ± 1.51	32.11 ± 6.96	34.29 ± 10.54	22.5 ± 3.8
Elastix	5.23 ± 1.49	53.27 ± 23.47	7.13 ± 2.03	5.45 ± 2.80	93.54 ± 1.12	5.33 ± 1.22	34.67 ± 12.85	38.42 ± 10.32	84.1 ± 7.2
VoxelMorph	5.89 ± 3.17	50.95 ± 28.90	7.06 ± 1.93	6.83 ± 2.85	93.18 ± 1.28	5.79 ± 1.64	35.88 ± 6.74	36.83 ± 11.77	0.20 ± 0.01
LapIRN	5.48 ± 2.24	52.51 ± 22.64	7.03 ± 1.71	5.51 ± 1.39	93.64 ± 1.13	5.52 ± 1.37	42.03 ± 4.71	49.03 ± 12.66	0.17 ± 0.02
macJNet	**5.05** ± **1.77**	**55.20** ± **18.77**	**6.71** ± **1.97**	**4.83** ± **1.49**	**94.75** ± **0.82**	**4.53** ± **1.11**	**44.12 ± 4.63**	**54.43** ± **11.62**	0.18 ± 0.02

Bold values indicate better results than other methods

#### Ablation studies

##### Evaluation of macMIND

To verify the effectiveness of our proposed macMIND in the macJNet, local mutual information (MI) [45], MIND [20] and macMIND are incorporated, respectively, into the macJNet to compare the performance of these modality-independent image descriptors. Table 3 shows the results of the competitive image descriptors for CT-MR deformable registration, which shows the proposed macMIND achieves the best performance in all evaluation metrics for global alignment and local deformation. macMIND have an ability to describes complex cross-modality image structures and their geometrical variants due to its multi-sampling patterns in self-similarity context. Moreover, macMIND also could robustly reflects the large deformation vis multi-scale sampling and cascaded extractions.

**Table 3:** Registration with different descriptors in joint learning framework (mean ± std)

Metric	Tumor			Liver			Image	
	TRE (mm)	DSC (%)	Hd_95_ (mm)	TRE (mm)	DSC (%)	Hd_95_(mm)	MI (%)	SSIM (%)
Affined	7.38 ± 3.56	46.81 ± 32.67	7.70 ± 3.27	7.01 ± 2.95	90.94 ± 1.38	6.67 ± 1.50	32.11 ± 6.96	34.29 ± 10.54
MI	6.03 ± 1.29	50.42 ± 25.68	7.33 ± 2.40	6.17 ± 2.24	92.89 ± 1.95	5.64 ± 1.12	43.65 ± 5.05	42.76 ± 9.78
MIND	5.64 ± 1.51	51.58 ± 23.05	7.18 ± 1.94	5.69 ± 1.41	94.61 ± 1.07	4.77 ± 1.08	42.72 ± 4.85	49.30 ± 12.67
macMIND	**5.05 ± 1.79**	**55.20 ± 18.77**	**6.71 ± 1.97**	**4.83 ± 1.49**	**94.75 ± 0.82**	**4.53 ± 1.11**	**44.12 ± 4.63**	**54.43 ± 11.62**

Bold values indicate better results than other methods

Compared with MIND in the joint learning framework, macMIND improves 10.34% for TRE, and 3.62% for DSC, and 6.59% for Hd_95_ in the local (tumor) registration; improves 15.05% for TRE, 0.14% for DSC, 5.03% for Hd_95_, and 5.13% for SSIM in organ (liver) alignment. The statistical results demonstrate that macMIND is an outstanding descriptor to represent modality-independent image structures.

Furtherly, the effectiveness of macMIND is evaluated in registration network with the unsupervised learning manner. The statistical results of macMIND and MIND are listed in Table 4. It is observed that macMIND significantly improves the performance of registration in almost all evaluation metrics. Specifically, macMIND improves 5.13% for TRE, 3.58% for DSC, and 7.07% for Hd_95_ in tumor registration; improve 10.97% for TRE and 2.36% for Hd_95_ in liver registration.

**Table 4:** Performance of macMIND in registration network (mean ± std)

Methods	Tumor			Liver			Image	
	TRE (mm)	DSC (%)	Hd_95_ (mm)	TRE (mm)	DSC (%)	Hd_95_(mm)	MI (%)	SSIM (%)
MIND	5.48 ± 2.24	52.51 ± 22.64	7.03 ± 1.71	5.51 ± 1.39	93.64 ± 1.13	5.52 ± 1.37	42.03 ± 4.71	49.03 ± 12.66
macMIND	**5.19 ± 1.34**	**56.09 ± 19.05**	**6.53 ± 2.18**	**4.90 ± 1.48**	93.64 ± 1.07	**5.39 ± 1.14**	**43.76 ± 4.83**	**53.00 ± 11.64**

Bold values indicate better results than other methods

**Fig. 3:**
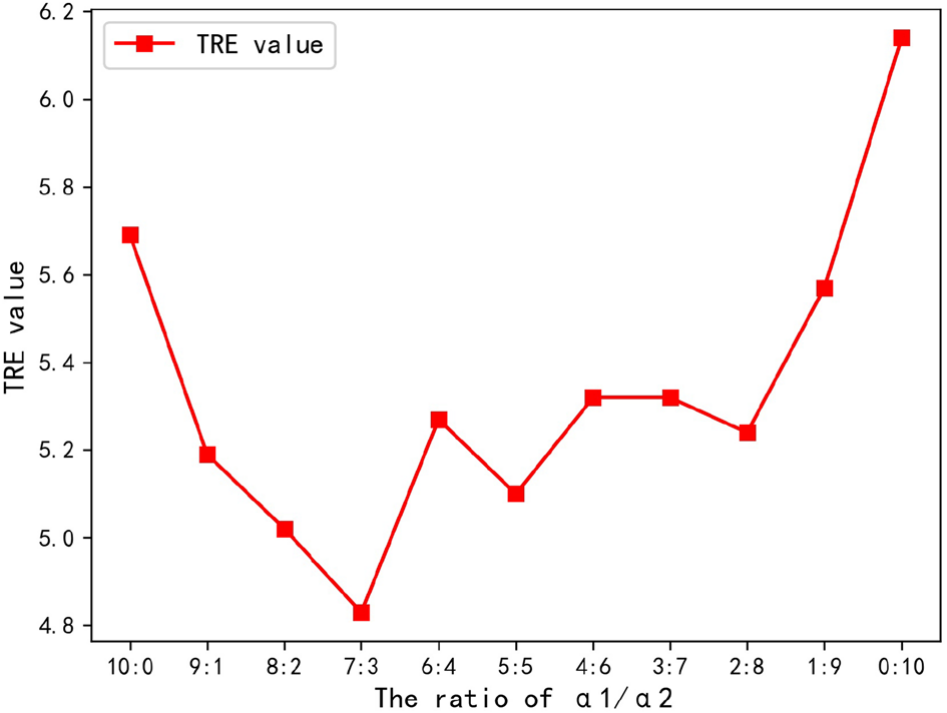
Optimal ratio of scale weight *α*_1_ and *α*_2_. The horizontal axis indicates the ratio of *α*_1_/*α*_2_, where α_2_ is the weight of large sampling window, and α_1_ is the weight of small sampling window

To further explore the influence of weight of self-similarity context in dual-scales, the optimal ratios of *α*_1_ and *α*_2_ is verified in the macJNet. Figure 3 gives an overview of different ratios of *α*_1_ and *α*_2_, which also demonstrates that *α*_1_/*α*_2_ = 7/3 is an optimal ratio value for CT-MR liver registration. In addition, the change of TRE values also illustrates the effectiveness of multi-scale sampling in macMIND.

##### Evaluation of joint learning framework

Organ labels of multimodal image pairs provide anatomical consistency constraint, which is considered as prior knowledge to guide alignment and deformation. However, manual labeling on multimodal images is a time-consuming task. Semi-supervised learning-based segmentation incorporated in a joint learning framework is a feasible way to provide segmentation labels for weakly-supervised registration. In this experiment, macJNet and Reg-SubNet is performed to access the effectiveness of anatomical consistency constraint. Reg-SubNet is considered as unsupervised registration network here since there are no inputting segmentation labels into it. macJNet also used 30% labels to training the Seg-SubNet, and macMIND is used as metric in macJNet and Reg-SubNet. The performance of macJNet and Reg-SubNet is listed in Table 5. The statistical result shows that the label-based anatomical consistency plays an important role in organ boundary alignment. It significantly improves the liver registration performance in this experiment: improving 1.43% for TRE, 1.19% for DSC, 15.96% for Hd_95_, 1.43% for SSIM. However, the influence of label-based anatomical consistency is diminished on the registration of internal lesion regions.

**Table 5:** Performance of macJNet and Reg-SubNet with macMIND (mean ± std)

Methods	Tumor			Liver			Image	
	TRE (mm)	DSC (%)	Hd_95_ (mm)	TRE (mm)	DSC (%)	Hd_95_(mm)	MI (%)	SSIM (%)
Reg-SubNet	5.19 ± 1.34	56.09 ± 19.05	6.53 ± 2.18	4.90 ± 1.48	93.64 ± 1.07	5.39 ± 1.14	43.76 ± 4.83	53.00 ± 11.64
macJNet	**5.05** ± **1.77**	55.20 ± 18.77	6.71 ± 1.96	**4.83 ± 1.49**	**94.75 ± 0.82**	**4.53 ± 1.11**	**44.12 ± 4.63**	**54.43 ± 11.62**

Bold values indicate better results than other methods

Some studies pointed out that the label-guided registration may receive diminishing or perturbing gradients [36, 37]. In the above experiment, DSC and Hd_95_ of the tumor are decreased due to the fact that the liver labels emphasize the alignment of the liver boundaries and ignores the physical properties of the deformation field, which yields some implausible deformation [31].

**Fig. 4:**
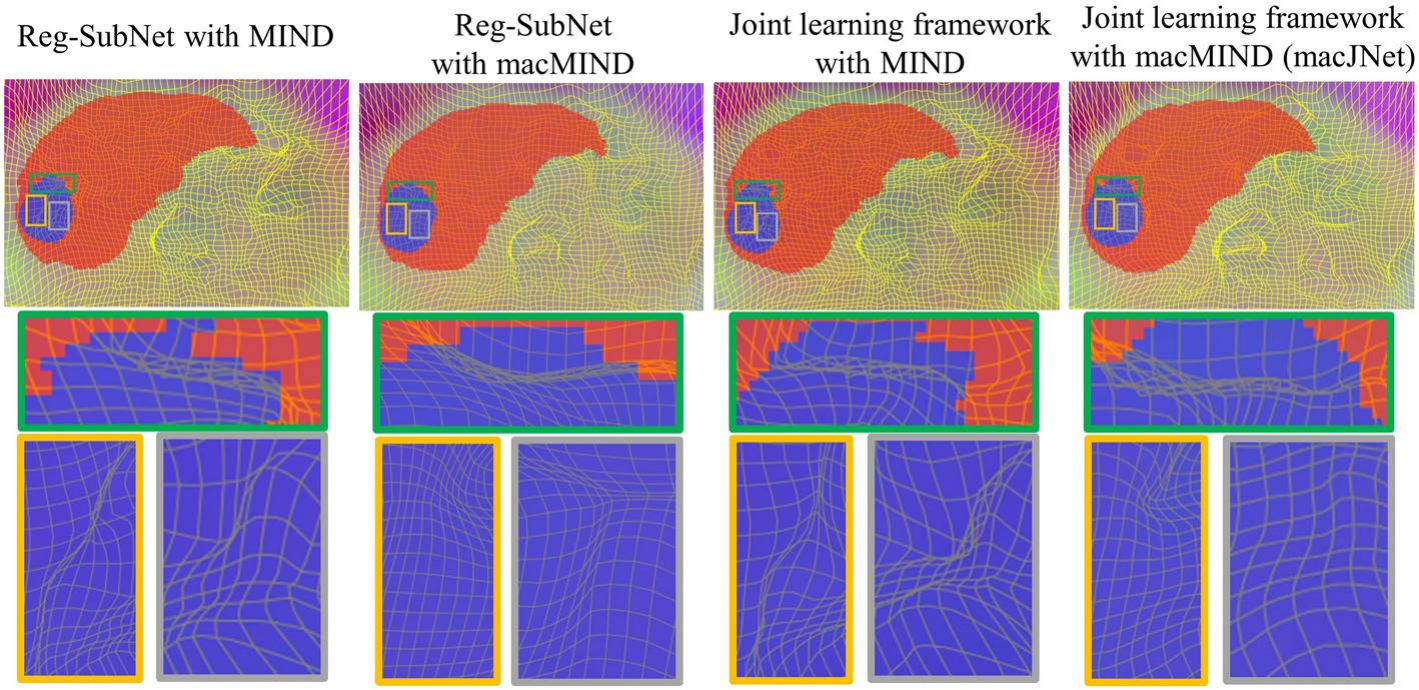
Visualization of registration on a sample in the test dataset. The four columns show the deformation field results of Reg-SubNet-MIND, Reg-SubNet-macMIND, JNet-MIND and macJNet in sequence. The red region is the liver label and the blue region is the tumor label

To investigate the effect of liver labels and modality-independent descriptors on the physical properties of the deformation field, the proportion of folding occurs (Jacobi determinant < 0) is calculated in different methods, as shown in Table 6. In the first set of experiments, the MIND descriptor and macMIND descriptor are separately applied to the Reg-SubNet (unsupervised registration). It is observed that macMIND performs significantly better than MIND with lower average proportion of folding points (0.63‱). In the second set of experiments, the two descriptors are applied separately to the joint learning framework (weakly-supervised registration), the average proportion of folding points in macMIND is also lower than that in MIND. It means that macMIND can effectively alleviates the negative impact of liver label and improve the physical properties of the deformation field. The visualization of deformation fields is shown in Fig. 4, which illustrates that macMIND effectively improves the physical properties of the deformation field.

**Table 6:** Performance of macMIND and MIND in deep learning-based registration (mean ± std)

Methods	det (*J*_*ϕ*_(*p*)) < 0 (‱)
Reg-SubNet with MIND	0.92 ± 0.45
Reg-SubNet with macMIND	**0.63 ± 0.22**
Joint learning framework with MIND	0.96 ± 0.47
Joint learning framework with macMIND (macJNet)	**0.74 ± 0.24**

Bold values indicate better results than other methods

**Fig. 5:**
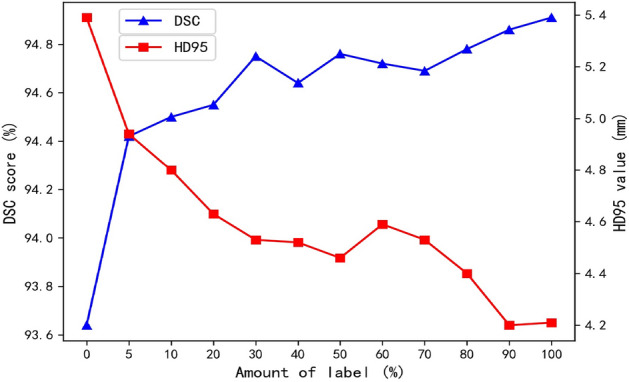
The influence of different number of labels on macJNet

Seg-SubNet is a semi-supervised segmentation network, which is influenced by the total amount of manual labeled images. To explore the influence of various amount of anatomy labels on registration, 0–100% different proportions of liver labels are input into Seg-SubNet by evaluating the registration metrics of liver registration. The results of liver registration are listed in Table 7. It obviously shows that the liver registration accuracy (DSC and H_d95_) gradually increases with the increase of label amounts, which demonstrates that the anatomy consistency of multimodal images provides prior knowledge to guide registration. The anatomy labels play an important role in alignment of organ boundaries: liver registration would be significantly improved if very few labels (such as 5% labels) are input into the joint learning registration framework. 30% of total amount of label would be considered as a trade-off between the time-consuming manual label task and registration accuracy, which can be seen clearly in Fig. 5.

**Table 7:** Registration results with different number of labels (mean ± std)

Amount	Liver			Image	
	TRE (mm)	DSC (%)	Hd_95_ (mm)	MI (%)	SSIM (%)
0%	4.90 ± 1.48	93.64 ± 1.07	5.39 ± 1.14	43.76 ± 4.83	53.00 ± 11.64
5%	4.93 ± 1.52	94.42 ± 0.85	4.94 ± 1.18	43.98 ± 4.70	53.72 ± 11.53
10%	5.18 ± 1.63	94.50 ± 0.92	4.80 ± 1.12	43.93 ± 4.60	54.41 ± 11.61
20%	5.24 ± 1.75	94.55 ± 0.91	4.63 ± 1.03	44.12 ± 4.58	54.85 ± 11.53
**30%**	**4.83 ± 1.49**	**94.75 ± 0.82**	**4.53 ± 1.11**	**44.12 ± 4.63**	**54.43 ± 11.62**
40%	4.79 ± 1.67	94.64 ± 0.82	4.52 ± 0.88	43.96 ± 4.55	54.22 ± 11.63
50%	5.05 ± 1.38	94.76 ± 0.85	4.46 ± 1.17	44.32 ± 4.64	54.39 ± 11.60
60%	5.10 ± 1.36	94.72 ± 0.89	4.59 ± 1.15	44.30 ± 4.71	54.05 ± 11.71
70%	5.27 ± 1.18	94.69 ± 0.86	4.53 ± 1.12	44.45 ± 4.68	54.24 ± 11.59
80%	5.31 ± 1.36	94.78 ± 0.79	4.40 ± 0.87	44.18 ± 4.72	54.17 ± 11.63
90%	4.97 ± 1.21	94.86 ± 0.89	4.20 ± 1.09	44.20 ± 4.52	53.94 ± 11.62
100%	5.10 ± 1.48	94.91 ± 0.81	4.21 ± 0.91	44.38 ± 4.65	54.09 ± 11.59

Bold values indicate better results than other methods

### Multimodal image segmentation results

Although our study aims to improve the performance of multimodal deformable registration, macJNet also have an ability to improve the performance of multimodal image segmentation due to its multi-modality consistency constraint for segmentation labels. macJNet provides consistency between labels by mapping the moving label to the fixed label via a deformation field.

To quantitatively verify the improvement of segmentation of macJNet, DSC, Hd_95_, recall, precision, absolute value of relative volume difference (RVD_abs_) and volumetric overlap error (VOE) are used to evaluate the segmentation accuracy. RVD_abs_ and VOE are defined as:

3 $${\text{RVD}}_{{{\text{abs}}}} = \left| {{{V_{{{\text{seg}}}} } \mathord{\left/ {\vphantom {{V_{{{\text{seg}}}} } {V_{{{\text{gt}}}} }}} \right. \kern-0pt} {V_{{{\text{gt}}}} }} - 1} \right| \times 100\% ,$$

4 $${\text{VOE}} = \left( {1 - {{\left( {V_{{{\text{seg}}}} \cap V_{{{\text{gt}}}} } \right)} \mathord{\left/ {\vphantom {{\left( {V_{{{\text{seg}}}} \cap V_{{{\text{gt}}}} } \right)} {\left( {V_{{{\text{seg}}}} \cup V_{{{\text{gt}}}} } \right)}}} \right. \kern-0pt} {\left( {V_{{{\text{seg}}}} \cup V_{{{\text{gt}}}} } \right)}}} \right) \times 100\% ,$$

where *V*_seg_ and *V*_gt_ symbol the segmentation volume and ground-truth volume, respectively.

The liver segmentation results on CT and MR images are listed in Table 8. Both macJNet and Sub-SegNet are trained with 30% labels. The statistical results of macJNet outperform the Sub-SegNet. Moreover, macJNet trained with 30% labels even surpasses the Sub-SegNet with 100% labels (DSC = 95.26, Hd_95_ = 8.71, Precision = 93.82, RVD_abs_ = 4.40, VOE = 9.04) for fixed (CT) image segmentation.

**Table 8:** Segmentation results on CT and MR with 30% labels (mean/std)

Modality	Methods	DSC (%)	Hd_95_ (mm)	Recall (%)	Precision (%)	RVD_abs_ (%)	VOE (%)
CT	Sub-SegNet	93.39 ± 3.31	19.15 ± 16.66	95.63 ± 6.24	91.50 ± 2.07	7.80 ± 4.25	12.24 ± 5.44
	macJNet	**95.38 ± 0.82**	**5.97 ± 3.21**	**96.05 ± 1.93**	**94.71 ± 1.24**	**2.53 ± 2.07**	**8.81 ± 2.19**
MR	Sub-SegNet	93.10 ± 2.39	20.95 ± 18.59	**95.23 ± 1.93**	91.20 ± 4.25	4.75 ± 6.22	12.81 ± 4.05
	macJNet	**94.12 ± 1.06**	**6.59 ± 2.14**	94.22 ± 2.13	**94.36 ± 2.72**	**3.92 ± 2.15**	**12.17 ± 1.86**

Bold values indicate better results than other methods

## Conclusion

This article has proposed macJNet for multimodal image deformable registration. macJNet is a weakly-supervised multimodal image deformable registration network using joint learning framework and macMIND. The main advantage of macJNet is that it provides global sparse correspondences by semantic labels and local dense correspondences by macMIND, where macMIND provides the local modality-independent contextual information. macJNet consists of a registration network and two segmentation networks. Each segmentation network generates semantic anatomical labels as weakly-supervised information for registration; macMIND incorporates multi-orientation and multi-scale sampling patterns to build self-similarity context, which is modality-independent image structure features and used as dense local contextual information to guide the registration. The registration network also provides the consistency of anatomical labels by spatial mapping for segmentation networks to improve the performance of multimodal image segmentation. Experiments on 3D CT-MR liver images have been carried out to evaluate performance of macJNet. Experimental results indicate that our method achieves significant improvements in multimodal registration task.

In future studies, label-efficient deep learning methods will be incorporated into our method to further reduce the reliance on manually labeled images. In addition, the impact of sampling scale number and multi-scale information fusion ways on registration results will be investigated.

## Methodology

### Overview

In this work, macJNet is proposed to improve the accuracy of multi-modality image registration. macJNet is a weakly-supervised multimodal image deformable registration method, which incorporates two components: joint learning framework and macMIND. The joint learning framework is a single end-to-end architecture, which includes two segmentation networks and a registration network. Segmentation networks provide semantic anatomical labels for weakly-supervised registration by few-label learning; registration network improves the performance of segmentation by enforcing cross-modality consistency based on deformable spatial mapping. macMIND builds the local self-similarity context by multi-orientation and multi-scale sampling in a supporting window, which enriches the modality-independence contextual information to characterize cross-modality anatomical structures. Detail of the proposed method is described in "Joint learning framework" and "Reg-SubNet and Seg-SubNet".

### Joint learning framework

**Fig. 6:**
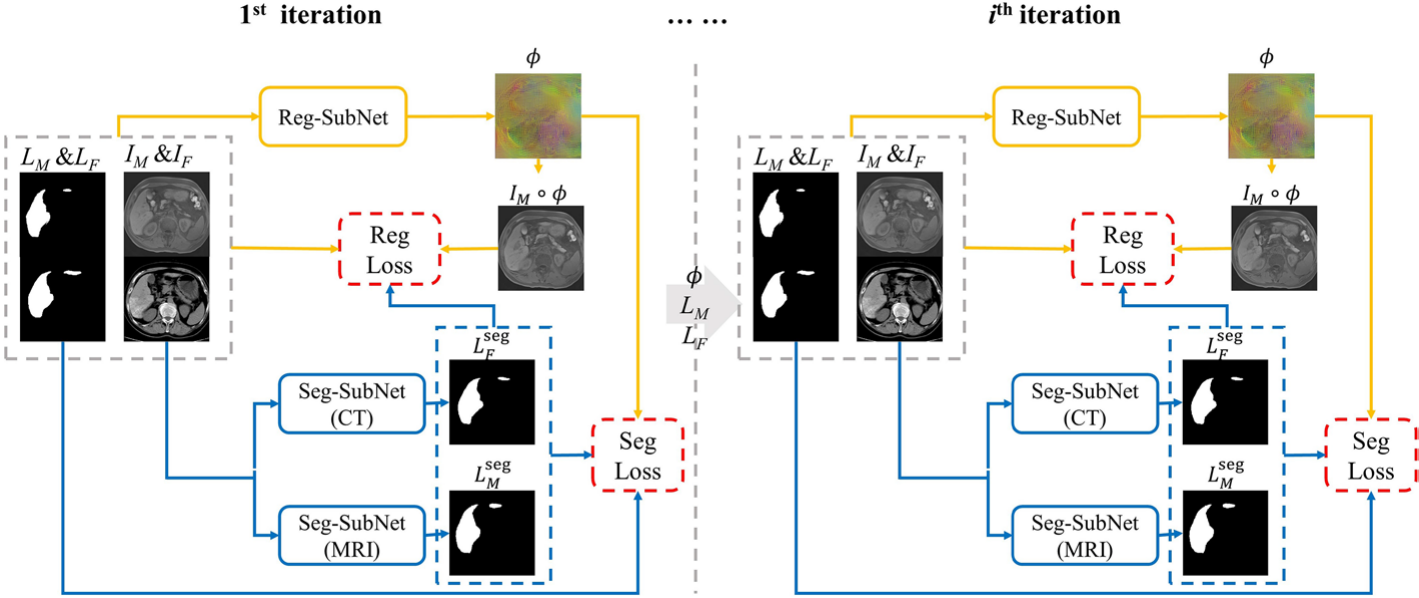
Illustration of macJNet for CT-MRI registration. Image labels *L* comprise two subsets: manual annotation label subset *L*^gt^ as ground-truth in segmentation network, prediction label subset *L*^seg^ is generated by Seg-SubNets. *L*_*M*_ = {*L*gt *M*, *L*seg *M*}, *L*_*F*_ = {*L*seg *F*, *L*seg *F*}. For each iteration, Reg-SubNet takes *I*_*M*_, *I*_*F*_ and their labels as input, outputs the deformation field *ϕ*, which provides the cross-modality consistency constrain for Seg-SubNets by mapping *L*_*M*_ to *L*_*F*_. Seg-SubNets take *I*_*M*_ and *I*_*F*_ as input, and output *L*seg *M* and *L*seg *F* to provide semantic labels as anatomical prior knowledge for registration

macJNet comprises three sub-networks, a weakly-supervised registration sub-network (Reg-SubNet) and two semi-supervised segmentation sub-network (Seg-SubNet) for dual-modality image segmentation. *K*_u_ unlabeled multimodal image pairs and *K*_l_ labeled image pairs (*K*_u_ > *K*_l_) are input into the network to optimize macJNet. Specifically, an alternately update strategy is used to optimize Reg-SubNet and Seg-SubNets in the joint learning framework. In the registration update stage, *I*_*F*_, *I*_*M*_ and their anatomy labels (including *K*_l_ pairs with manual labels *L*^gt^ and *K*_u_ pairs with segmentation labels *L*^seg^ created by Seg-SubNets) are input into Reg-SubNet to optimize the dense deformation fields *ϕ*. In the segmentation update stage, *K*_u_ unlabeled image pairs and *K*_l_ labeled image pairs are input into the Seg-SubNets to generate the segmentation labels (*L*seg *M* and *L*seg *F*), where the dense deformation fields created by Reg-SubNet maps *L*seg *M* to *L*seg *F* for cross-modality consistency constraint. The overview of the joint learning framework is illustrated in Fig. 6.

The main advantages of joint learning in macJNet are as follows: (1) incorporating two correlated tasks in a single framework to improve the performance of registration; (2) allowing to use existing task-specific networks for registration and segmentation. It is noteworthy that our work does not focus on the design of an elaborate registration network. The main aim of this work is to propose a general framework for weakly-supervised registration, any task-specific registration or segmentation networks could be used as sub-networks in this framework. Some other works [27, 38] joint the registration and segmentation through multi-task learning. Multi-task learning methods joint the two tasks using hard or soft parameter sharing, which needs to change the architecture of existing networks.

### Reg-SubNet and Seg-SubNet

**Fig. 7:**
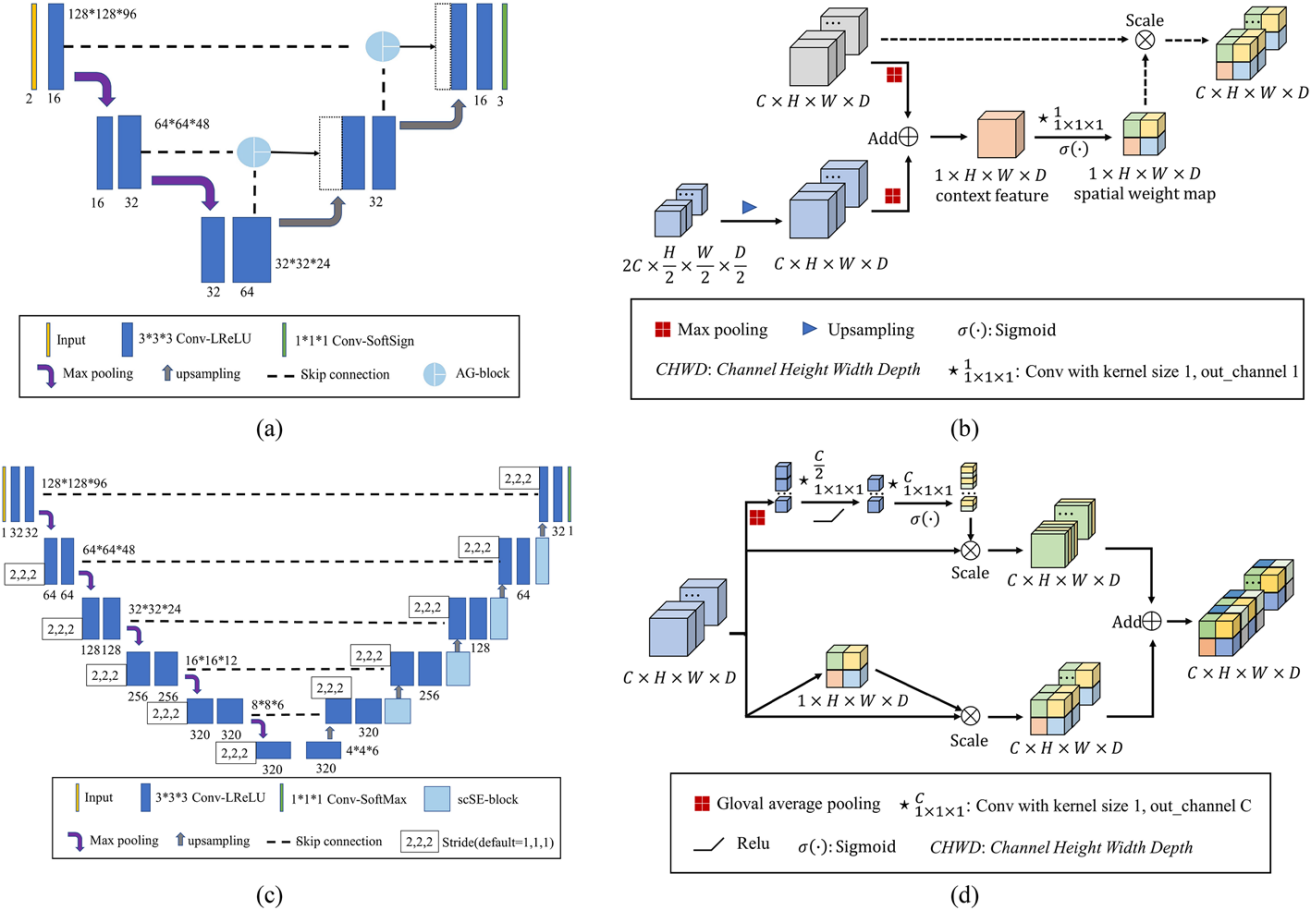
Reg-SubNet and Seg-SubNet in the joint learning framework. **a** The architecture of Reg-SubNet; **b** AG-block in Reg-SubNet; **c** architecture of Seg-SubNet. **d** scSE-block in Seg-SubNet

In this study, LapIRN is adopted to build Reg-SubNet (shown in Fig. 7a). LapIRN [35] is a deep Laplacian pyramid image registration network with a 3D UNet-like architecture [39] and mitigates the large-deformation problem via a coarse-to fine scheme [35]. AG-blocks [40] (shown in Fig. 7b) are added into the LapIRN to filter the features by propagating through the skip connections. AG-blocks employ multi-level spatial and contextual information to highlight the regions with large discrepancies. The nnUNet [41] is applied to build Seg-SubNet (shown in Fig. 7c) due to its excellent performance in medical image segmentation. scSE-blocks [42] (shown in Fig. 7d) are added into the decoding layers to suppress insignificant information in both spatial and channel dimensions. In the training stage, Dice loss is used to measure the similarity in label pairs.

### Multi-sampling cascaded modality independent neighborhood descriptor

#### Modality independent neighborhood descriptor

MIND is a well-known image representor [20] for multi-modality image registration, which represents local self-similarity structures by calculating the difference between patches within a local neighborhood. For any point *x* in image *I*, the MIND feature can be represented by Gaussian kernel distance between center point *x* and its 6-neighborhood patches, as shown in Fig. 9b. Assuming that the *n*-th patch in the 6-neighborhood centered at *x*_*n*_, MIND can be expressed as:

5 $${\text{MIND}}\left( {I,x,x_{n} } \right) = \exp \left( { - \frac{{D_{p} \left( {I,x,x_{n} } \right)}}{{V\left( {I,x} \right)}}} \right)$$

where *D*_*p*_(*I*, *x*, *x*_*n*_) donates the mean squared difference between two patches, which, respectively, locate at* x* and *x*_*n*_. *P* is defined as the set of displacements from any voxel in a patch to the center of the patch.

6 $$D_{p} \left( {I,x,x_{n} } \right) = \frac{1}{\left| P \right|}\sum\limits_{t \in P} {\left( {I\left( {x_{{}} + t} \right) - I\left( {x_{n} + t} \right)} \right)^{2} } ,$$

*V*(*I*, *x*) is an estimation of the local variance, defined as the expectation of *D*_*p*_:

7 $$V\left( {I,x} \right) = \frac{1}{6}\sum\limits_{n = 1}^{6} {D_{p} \left( {I,x,x_{n} } \right)} .$$

However, MIND computes self-similarity between the center patch and its 6-neighborhood patches with the simple sampling pattern (shown in Fig. 9), which cannot handle the large deformation and high complex dense correspondence.

#### macMIND

Inspired by MIND, a multi-sampling cascaded modality independent neighborhood descriptor (macMIND) is proposed to improve performance of multimodal image deformable registration. The motivation of macMIND is to incorporate more abundant sampling patterns for representing the complex cross-modality structure features, which contributes to find dense correspondence in multimodal images.

**Fig. 8.**
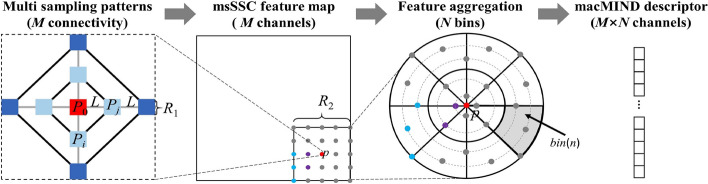
2D illustration of cascaded feature calculations of macMIND. The “Multi sampling partners” sketch illustrates the msSSC with multi-sampling patterns (multi-scale sampling and multi-orientation sampling). msSSC includes some different scale self-similarity contexts (SSC). The left sketch illustrates a dual-scale SSC: the small-scale SSC includes the central patch *P*_0_ (red box) and its closer 4-neighborhood (light blue boxes); the larger-scale SSC includes the central patch *P*_0_ and its farther 4-neighborhood (dark blue boxes). Each SSC includes more connectivity (black lines and gray line) than MIND (gray lines), which leads macMIND to incorporate more orientation sampling. *L* and *R*_1_ symbolize the patch distance and size, respectively. The msSSC feature map with *M* channels are created. The “feature aggregation” sketch shows the *N* bins (here *N* = 16) in log-polar space with 8-angle intervals and 2-radial intervals. One of the bins is colored with gray. macMIND translates each voxel in an image to a *M* × *N* matrix by macMIND. Finally, macMIND feature map is created as a *M* × *N* channel image for registration

The macMIND descriptor incorporates cascaded feature calculations: (1) multi-scale self-similarity context (msSSC) feature map calculation with multi-sampling patterns; (2) feature aggregation in 3D log-polar bins. Figure 8 illustrates the cascaded feature calculations of macMIND. macMIND extracts the *M* × *N*-channels feature map of every voxel in the image. The specific implementation process and its advantages will be detailed in the following sections.

#### Multi-sampling patterns of msSSC

**Fig. 9:**
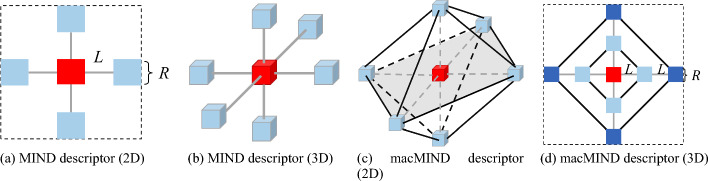
Illustration of MIND and macMIND descriptor. **a** The dotted box illustrates the supporting window, the red box illustrates the central patch of the supporting window, its 6-neighborhood patches are colored in blue.* L* and *R* symbolize the patch distance and size, respectively. **b** The 3D structure of 6-connectivities (3-orientation) in MIND. **c** The single scale 3D structure layout of 18-connectivities (9-orientation) in macMIND. The black (shown as black solid lines and black dotted lines) 12-connectivities are introduced by macMIND, while the gray 6-connectivities (3-orientation) attached to MIND. **d** The supporting window of macMIND similar with MIND. Light blue and dark blue patches indicate the dual sampling scales, and black and gray connections indicate the multi sampling orientation

Multi-sampling patterns (multi-orientation sampling and multi-scale sampling) are introduced to encode the self-similarity context, which is robust and accurate cross-modality feature representation. Specifically, given a certain patch layout *P*_Ω_, the central patch *P*_0_ of size *R*_1_ × *R*_1_ × *R*_1_ centered at voxel *p* and the distance between *P*_0_ and its 6-neighborhood patches is *L* (Fig. 8a). The self-similarity context *SSC*(*I*, *P*_Ω_) is defined as:

8 $${\text{SSC}}\left( {I,P_{\Omega } } \right) = \sum\limits_{{P_{i} ,P_{j} \in P_{\Omega } }} {\exp \left( { - \frac{{\left\| {e_{P} } \right\|SSD\left( {I,P_{i} ,P_{j} } \right)}}{{\sum\limits_{{P_{i} ,P_{j} \in P_{\Omega } }} {SSD\left( {I,P_{i} ,P_{j} } \right)} }}} \right)} ,$$

where *I* is an image, *P*_Ω_ = {*P*_0_, *P*_1_,…,*P*_6_}, *P*_*i*_ and *P*_*j*_ are the symbols of arbitrary patches in *P*_Ω_, ∥*e*_*P*_∥ denotes the total number of patch connections. *SSD*(*I*, *P*_*i*_, *P*_*j*_) denotes the sum of squared difference between patch *P*_*i*_ and *P*_*j*_, which is formulated as:

9 $${\text{SSD}}\left( {I,P_{i} ,P_{j} } \right) = \frac{1}{\left\| P \right\|}\left( {I\left( {P_{i} } \right) - I\left( {P_{j} } \right)} \right)^{2} ,$$

where ∥*P*∥ represents the total number of voxels in patch *P.* In Eq. (8), SSC is computed as the sum of squared difference between two patches to represent the self-similarity. As shown in Fig. 9d, there are 18-connectivity in SSC within single scale, which can be divided into 9 orientations. Therefore, a multi-orientation sampling pattern is introduced into macMIND, which leads macMIND to represent the complex deformations.

The multi-scales self-similarity context (msSSC) is further computed to represent the large deformation (large geometrical variations) in the multimodal images. msSSC can be reformulated as:

10 $${\text{msSSC}}\left( p \right) = \sum\limits_{k = 1}^{K} {\alpha_{k} } {\text{SSC}}\left( {I,P_{{_{\Omega } }}^{k} } \right) = \sum\limits_{k = 1}^{K} {\alpha_{k} \sum\limits_{{P_{i} ,P_{j} \in P_{{_{\Omega } }}^{k} }} {\exp \left( { - \frac{{\left\| {e_{P} } \right\|{\text{SSD}}\left( {I,P_{i}^{k} ,P_{j}^{k} } \right)}}{{\sum\limits_{{P_{i} ,P_{j} \in P_{{_{\Omega } }}^{k} }} {{\text{SSD}}\left( {I,P_{i}^{k} ,P_{j}^{k} } \right)} }}} \right)} } ,$$

where *K* is the total number of scales, and *α*_*k*_ denotes the weight of multi-self-similarity context in the *k*-th scale. The Eq. (10) formulates the multi-self-similarity context in a weighted sum way. It is a simple way to fuse the multi-scale information in consideration of the limitations of computing resources. The other way is to concatenate *SSC*(*I*, *P*_Ω_) of each scale along the channel dimension.

In summary, self-similarity in MIND is calculated based on the central patch (shown in Fig. 9a, b), which has the disadvantage that the noise in central patch takes adverse effect on the self-similarity. Compared with MIND, msSSC has two advantages: (1) multi-orientation sampling: utilizing all pairwise connectivity (18- connectivity) within central patch and its 6-neighbourhood to build a 9-orientation sampling pattern (shown in Fig. 9c); (2) multi-scale sampling: incorporating multi-scale self-similarity context in a supporting window (shown in Fig. 9c). The multi-sampling pattern in msSSC leads macMIND to represent the complex and large deformation in multimodal images.

#### Feature aggregation in 3D log-polar bins

Each point in the msSSC feature map is aggregated into the log-polar bins [24] to robustly represent the cross-modality structural information in deformable registration [43, 44]. A patch with size *R*_2_ × *R*_2_ × *R*_2_ and central at voxel *p* on msSSC is selected, and all voxels in the patch are transformed into local 3D log-polar space. The 3D log-polar space is divided into *N* bins based on *N*_*a*_ angle intervals, *N*_*r*_ radial intervals and *N*_*h*_ height intervals (*N* = *N*_*a*_ × *N*_*r*_ × *N*_*h*_). The values in each bin are calculated and the average values are concatenated into a *M* × *N*-dimension vector as a macMIND descriptor. The macMIND is defined as

11 $${\text{macMIND}}\left( {I,p} \right) = \mathop {{\text{cat}}}\limits_{{n \in \left\{ {1,2 \cdots N} \right\}}} \left( {\mathop {{\text{average}}}\limits_{p \in bin\left( n \right)} \left( {{\text{msSSC}}\left( p \right)} \right)} \right),$$

where ‘cat’ symbolizes the vector concatenation. Finally, macMIND translates each voxel *p* in image to a *M* × *N* vector. Here, the msSSC feature image is aggregated by utilizing average pooling in bins instead of max-pooling to maintain the fine-scale matching details [45].

**Fig. 10:**
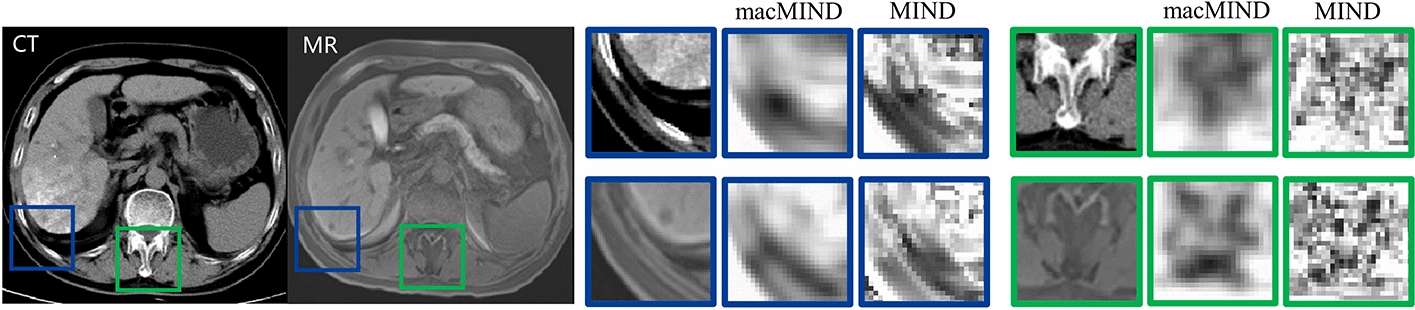
Feature map visualization of macMIND and MIND. macMIND shows its advantage for representing complex anatomical structures. The multi-channel feature map is translated to a single-channel image by calculating the average value of channel-dimension for visualization. In this figure, macMIND is calculated with *K* = 2, *α*_1_ = 0.7, *α*_2_ = 0.3

Figure 10 shows the comparisons of feature map between macMIND and MIND on CT-MRI images. Two typical locations with different image structures are selected: (1) the boundary between liver and abdomen (blue window), it is a latent region with large deformation; (2) spine (green window), it is a region with complex structures. It is obviously observed that the macMIND feature map accurately represents the modality-independent features (e.g., tissue boundary and shape), and is more continuous and smoother than MIND.

#### Cascaded feature extraction

**Fig. 11:**
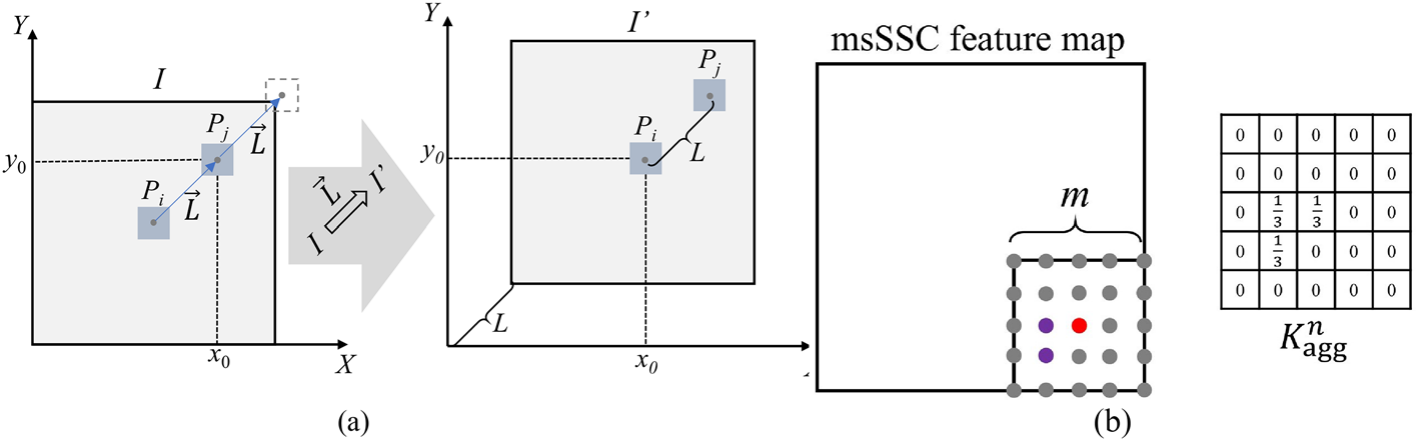
The convolution operations in SSD calculation and feature aggregation. **a** SSC feature map is calculated in the manner of convolution operations. *P*_*i*_ is the center patch of the image, *P*_*j*_ is a neighborhood patch of *P*_*i*_. *P*_*i*_ would overlap *P*_*j*_ by shifting with $\overrightarrow {L}$. This operation translates the computation of SSD to a voxel-wise squared difference. **b** Feature aggregation with a convolution operation. The kernels are designed according to the spatial distribution of voxels in bins

Actually, SSD is computed and feature map aggregation is performed in a convolutional manner due to its computational efficiency. Specifically, for computing *SSD*(*I*, *P*_*i*_,* P*_*j*_), image *I'* is obtained by shifting the image *I* by a vector $\overrightarrow {L}$, as shown in Fig. 11a. *I*(*P*_*j*_) is equal to *I*'(*P*_*i*_) since the distance between patch *P*_*i*_ and patch *P*_*j*_ is $\left\| {\overrightarrow {L} } \right\|$. Then, the voxel-wise squared difference is calculated in the minus manner of *I* and *I'*: *I*(*P*_*i*_)-*I*(*P*_*j*_) = *I*(*P*_*i*_)-*I*'(*P*_*i*_). Finally, the patch-wise squared difference can be obtained from voxel-wise squared difference by convolution with a *R*_1_ × *R*_1_ × *R*_1_ sized kernel *K*_SSD_. *K*_SSD_ is designed as an average pooling kernel. The *SSD*(*I*, *P*_*i*_, *P*_*j*_) computation in Eq. (9) can be effectively substitute, which is reformulated as:

12 $${\text{SSD}}\left( {I,P_{i} ,P_{j} } \right) = K_{{{\text{SSD}}}} \otimes \left( {I - I{\prime} } \right)^{2} ,$$

where ⊗ is the convolution operator. For aggregating the point of msSSC feature map in 3D log-polar bins, a specific convolution kernel *Kn* agg (*n* is the scale parameter in msSSC, *n* = 1,2,…,*N*) with size of *R*_2_ × *R*_2_ × *R*_2_ is designed on the msSSC feature map (shown in Fig. 11b) for each bin. *Kn* agg transforms the mean value calculation to a convolution operation. The Eq. (11) could be reformulated as

13 $${\text{macMIND}}\left( {I,p} \right) = \mathop {{\text{cat}}}\limits_{{n \in \left\{ {1,2 \cdots N} \right\}}} \left( {K_{{{\text{agg}}}}^{n} \otimes {\text{SSC}}\left( p \right)} \right),$$

**Fig. 12:**
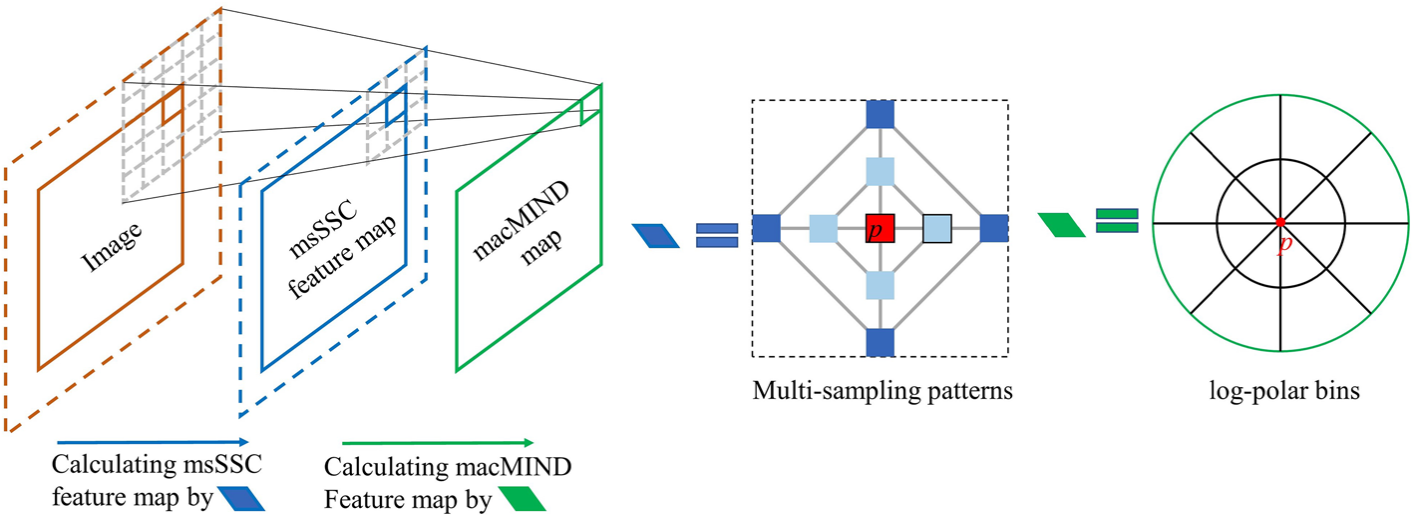
Illustration of cascaded convolution operations in macMIND

The cascade convolution operations of macMIND are similar to the feature learning in two consecutive encoder layers of CNN (shown in Fig. 12), which has two advantages for registration: representing deeper and more complex features, enlarging the receptive field of feature representation with low computational cost [24].

The sampling density is a main difference between MIND [21], macMIND, and DSC [24]. On one hand, compared with MIND, macMIND increases the sampling density by utilizing all connectivity of patch layout to encode comprehensive information in SSC feature map. All connectivity of patch layout also introduces multi-scale and multi-orientation sampling patterns. The increase of sampling density enriches the modality-independence contextual information for dense correspondence cross multimodal images. On the other hand, in comparison to the deep learning-based sampling on the self-similarity surface [24], macMIND supplies a sparse sampling with the fixed patterns. Although some dense sampling patterns have been proposed to build more elaborate cross-modality descriptors (such as DSC [24] and DASC [45]), they would be computationally intractable for 3D medical images. The sparse sampling patterns are necessary for efficient computation in 3D medical image registration. The patch layout in the supporting window of macMIND is much sparser than the dense self-correlation surfaces in DSC and DASC.

### Loss function in joint learning framework

Dual-similarity-based loss is proposed for registration: a macMIND-based similarity metric to capture the dense correspondence of modality-independent texture characteristics, DSC-based similarity to capture the semantic consistency of anatomical characteristics in multimodal images. DSC value of label images is used as loss to guide the Seg-SubNet training.

#### Dual similarity-based loss for multimodal image registration

The loss function for Reg-SubNet is defined as: *E*_reg_ = *E*_*sim*_(*I*_*F*_, *I*_*M*_∘*ϕ*) + *λ*_label_*E*_label_(*L*_*F*_, *L*_*M*_∘*ϕ*) + *λ*_smo_*E*_smo_(*ϕ*). *E*_sim_(*I*_*F*_, *I*_*M*_∘*ϕ*) takes the form as

14 $$E_{{{\text{sim}}}} (I_{F} ,I_{M} \circ \phi ) = \frac{1}{{\left| {\Omega^{3} } \right|}}\sum\limits_{{p \in \Omega^{3} }} {\left( {{\text{macMIND}}\left( {I_{F} ,p} \right) - {\text{macMIND}}\left( {I_{M} \circ \phi ,p} \right)} \right)}^{2} ,$$

*E*_sim_ measures the local difference between the pair of macMIND maps, |Ω^3^| is the total voxel number of the image. *E*_label_ measures the DSC value of fixed label image and warped label image. In addition, a diffusion regularization on the spatial gradients of *ϕ* is added to encourage a smooth deformation field, which is defined as

15 $$E_{{{\text{smo}}}} \left( \phi \right){ = }\sum\limits_{{p \in \Omega^{3} }} {\left\| {\nabla \phi \left( p \right)} \right\|}^{2} .$$

#### Loss function for semi-supervised segmentation

The loss function for Seg-SubNet is defined as:

16 $$\left\{ \begin{array}{lll}E_{{{\text{seg}}}} = E_{{{\text{DSC}}}} \left( {L_{F}^{{{\text{seg}}}} ,L_{M}^{{{\text{seg}}}} \circ \phi } \right) & \quad {L^{{{\text{gt}}}} \;{\text{not}}\;{\text{existed}}}\\E_{{{\text{seg}}}} = E_{{{\text{DSC}}}} \left( {L_{F}^{{{\text{seg}}}} ,L_{F}^{{{\text{gt}}}} } \right) + E_{{{\text{DSC}}}} \left( {L_{M}^{{{\text{seg}}}} ,L_{M}^{{{\text{gt}}}} } \right) & \quad {L^{{{\text{gt}}}} \;{\text{existed}}} \end{array} \right.$$

*L*^seg^ represents the segmentation label output by Seg-SubNet, *L*^gt^ represents the ground-truth. *E*_DSC_(*L*^seg^, *L*^gt^) guides the segmentation results to the ground-truth, and *E*_DSC_(*L*seg* F*, *L*seg* M*∘*ϕ*) guides different Seg-SubNets to generate consistent segmentations labels.

## Data Availability

Not applicable.
